# Multiplex Profiling of Cellular Invasion in 3D Cell Culture Models

**DOI:** 10.1371/journal.pone.0063121

**Published:** 2013-05-09

**Authors:** Gerald Burgstaller, Bettina Oehrle, Ina Koch, Michael Lindner, Oliver Eickelberg

**Affiliations:** 1 Comprehensive Pneumology Center, University Hospital of the Ludwig-Maximilians-University Munich and Helmholtz Zentrum München, Member of the German Center for Lung Research, Munich, Germany; 2 Center for Thoracic Surgery, Asklepios Biobank for Lung Diseases, Comprehensive Pneumology Center, Asklepios Clinic Munich-Gauting, Munich, Germany; University of Tübingen, Germany

## Abstract

To-date, most invasion or migration assays use a modified Boyden chamber-like design to assess migration as single-cell or scratch assays on coated or uncoated planar plastic surfaces. Here, we describe a 96-well microplate-based, high-content, three-dimensional cell culture assay capable of assessing invasion dynamics and molecular signatures thereof. On applying our invasion assay, we were able to demonstrate significant effects on the invasion capacity of fibroblast cell lines, as well as primary lung fibroblasts. Administration of epidermal growth factor resulted in a substantial increase of cellular invasion, thus making this technique suitable for high-throughput pharmacological screening of novel compounds regulating invasive and migratory pathways of primary cells. Our assay also correlates cellular invasiveness to molecular events. Thus, we argue of having developed a powerful and versatile toolbox for an extensive profiling of invasive cells in a 96-well format. This will have a major impact on research in disease areas like fibrosis, metastatic cancers, or chronic inflammatory states.

## Introduction

Intravasation and/or transmigration of individual or collective cells in tissues is the hallmark of diseases like metastasis, fibrosis, or chronic inflammation [1]. Elucidating the underlying mechanisms of aberrant cellular invasion in tissues is therefore crucial and fundamental for the therapeutic targeting of above-mentioned diseases. Thus far, monotherapies of possible targets interfering with the migration of cells throughout an extracellular matrix (ECM), like matrix metalloproteases (MMPs), were staggeringly inefficient, though a combination of inhibitors still holds a promising outlook [2]. Therefore, the accelerated high-throughput screening of therapeutic compounds interfering with cellular invasion along with an efficient multiparametric high-content analysis at a minimum cost becomes a highly preferable goal [2]. However, most migration and invasion assays exist only for conventional 2D cell culture techniques that in fact cannot closely mimic the complex mechanical and biochemical interplay between various cells and their ECM microenvironment in real tissue. An informative description of a wide variety of commonly applied three-dimensional (3D) invasion assays has recently been reviewed [3], though none of the described assays can meet the above mentioned criteria at the same time.

Culturing cells on planar plastic or glass support has led to a plethora of studies investigating and understanding cell migration in two dimensions (2D). Nevertheless, an increasing amount of publications reveals considerable morphological and functional diversities by culturing cells in 3D-ECM microenvironments. Variations in gene-expression patterns, cell morphology, cellular differentiation, cell-matrix adhesions and migration were reported [4]–[9]. Intriguingly, cells may likewise switch between integrin-dependent and integrin-independent modes of migration in 3D microenvironments [10]. 3D tissue cultures are thought to more closely resemble the in vivo situation of animal or human tissues regarding composition and stiffness of the matrix [6]. Importantly, 3D tissue culture conditions are of relevance for in vitro experiments with cells like pericytes or fibroblasts that usually appear in interstitial compartments. Therefore, 3D tissue culture bears biological advantages in mimicking a more physiological in vivo situation, leading to a better translation of ground-breaking findings in basic research to the clinic. However, using 3D ECM microenvironments adds a higher level of complexity and therefore bears numerous technological challenges in respect of cell culture, immunohistochemistry and image acquisition.

To address invasion dynamics and molecular signatures thereof in a high-content fashion, we have developed an inexpensive, multiparametric, 96-well-microplate-based, 3D cell culture assay. Our assay is capable of combining dimensions of cell motility and invasion, together with cell-morphology and biomarkers. Additionally, we can correlate mRNA and protein signatures to invasion by utilizing a slightly modified version of the invasion assay. Above all, our technique is suitable for pharmacological screening of novel compounds regulating invasive and migratory pathways of primary cells in a 96-well plate format.

## Materials and Methods

### Ethics Statement

Resected human lung tissue was used for isolation of primary cells. Participants provided written informed consent to participate in this study, in accordance with approval by the local ethics committee of the LMU (Ludwig-Maximilians Universität) of Munich, Germany (Project 333-10).

### Antibodies

For immunofluorescence microscopy the following primary (1) and secondary (2) antibodies (Abs) were used: 1) rat monoclonal Abs to Ki67 (Dako, 1∶100) and to CD29 (9EG7, BD Pharmingen, 1∶100), goat polyclonal Ab to vimentin (C20, Santa Cruz, 1∶100), and rabbit polyclonal Ab to fibronectin (H-200, Santa Cruz, 1∶100); and 2) donkey anti-goat IgG Alexa Fluor 488 (Invitrogen), goat anti-rat IgG Alexa Fluor 488 (Invitrogen), and goat anti-rabbit IgG DyLight^TM^649 (Jackson ImmunoResearch Laboratories, Inc.). Rhodamine Phalloidin (Life Technologies) was used in a dilution of 1∶200. Cell nuclei were stained with DAPI (4′,6-diamidino-2-phenylindole, Sigma) in a dilution of 1∶2000. For immunoblotting MMP13 (ab75606, Abcam, 1∶333) and monoclonal mouse anti-β-Actin-Peroxidase (AC-15, Sigma, 1∶10000) were used as primary antibodies, and goat anti-rabbit IgG conjugated to horseradish peroxidase (Cell Signaling, 1∶10000) as secondary antibody.

### 3D Collagen Gels, Cell Culture and Transient Transfections

To generate 3D collagen gels, collagen G (L1613, Biochrome), produced from calf skin, was used according to the manufacturer’s instructions. In brief, solution A was prepared by mixing 1M HEPES buffer (Sigma) and 0.7M NaOH in a 1∶1 ratio. Mixing solution A with 20% FBS (PAA) in 10× PBS (pH = 7.4) in a 1∶1 ratio resulted in solution B (pH = 7.90–8.05). For the final gelation collagen G and solution B were mixed in a 4∶1 ratio. In order to get a homogenous gelation it is crucial to keep all reagents on ice (4°C). 40 µl of collagen G/solution B mixture were added to each well of a tissue culture treated 96-well microplate with a flat bottom (catalog# 353376, BD Biosciences). Putting 40 µl of collagen G/solution B per well resulted in a 200–300 µm thick polymerized 3D collagen matrix, whereas less volume gave bad results in the overall quality of the polymerized 3D collagen matrix. Using a higher volume than 40 µl restricts the usage of objectives higher than 10× during image acquisition. To allow gelation of the collagen G the 96-well microplate was incubated at 37°C for 1 hours and the quality of the polymerized collagen gels was checked with an Axiovert 40C microscope (Zeiss). Mouse lung fibroblasts MLg (Mlg 2908) were purchased from ATCC (CCL-206) and cultivated in DMEM/HAM’s F12 (catalog# E15-813, PAA) medium containing 10% FBS. Cells were cultivated and passaged under standard conditions (5% CO_2_ and 37°C) and were not used at passage numbers higher than 15. Cells were transiently transfected in DMEM/HAM’s F12 medium containing 10% FBS using Lipofectamine™ 2000 (Invitrogen) in a 6-well format according to the manufacturer’s manual. Experiments were carried out 16–24 h after transfection.

### Isolation of Primary Human Fibroblasts

Specimens from lung lobes or segmental lung resections were dissected into pieces of 1–2 mm^2^ in size and digested by 5 mg of Collagenase I (Biochrom) at 37°C for 2 hours. Subsequently, samples were filtered through nylon filters with a pore size of 70 µm (BD Falcon). Filtrates, containing the cells, were centrifuged at 400 g, 4°C for 5 minutes. Pellets were resuspended in DMEM/F-12 medium (Gibco) supplemented with 20% fetal bovine serum (PAA) and plated on 10 cm cell-culture dishes. Medium was changed after 2 days and cells were split after reaching a confluence of 80–90%.

### Confocal Live Cell Imaging

Confocal time-lapse microscopy was implemented on an LSM710 system (Carl Zeiss) containing an inverted AxioObserver.Z1 stand. 3D collagen matrices containing 5 ng/ml TGFβ_1_ were cast in 3.5 cm cell culture dishes. Cells were stained with 5 µM of the red cell-tracker dye CMTPX (Molecular Probes) and then seeded on top of the 3D collagen matrix. Cells were kept in DMEM/HAM’s F12 medium containing 10% FBS and 15 mM HEPES during the whole period of observation. Imaging of the cells was performed in a PM S1 incubator chamber (Carl Zeiss) at 37°C. Recordings started within one hour after seeding the cells on top of the collagen matrix, and z-stacks (245 µm) were acquired with an EC Plan-Neofluar DICI 10x/0.3 NA objective lens (Carl Zeiss) in 30 minutes intervals operated by ZEN2009 software (Carl Zeiss). The settings for the LSM were as follows: zoom = 0.6, pixel dwell time = 3.15 µs, average = 1, master gain = 899, digital gain = 1.00, digital offset = 0.00, pinhole = 90 µm, filters = 410–556, laser line 561 nm = 2%. The confocal 4D data sets were imported into Imaris 7.4.0 software (Bitplane) and processed by applying a maximum intensity projection volume rendering algorithm.

### 3D Collagen Invasion Assay, Confocal Imaging and Data Analysis

After gelation of the 3D collagen gel 2×10^4^ cells per well were seeded on top of the matrix and left for invasion under standard conditions (37°C, 5% CO_2_) in DMEM/HAM’s F12 medium containing 1% FBS for 72 hours. Treatment of cells with epidermal growth factor (EGF) was accomplished by culturing cells in DMEM/HAM’s F12 medium containing 1% FBS supplemented with 50 ng/ml EGF. The collagen gels containing the cells were washed once in PBS (138 mM NaCl, 26 mM KCl, 84 mM Na_2_HPO_4_, 14 mM KH_2_PO_4_, pH 7.4), fixed in 4% paraformaldehyde (PFA) in PBS for 1 hour and permeabilized in 4% PFA/0.5% Triton X-100 in PBS for 10 minutes. Subsequently, the nuclei of cells were stained with DAPI (1∶2000) in PBS for 1 hour at room temperature. Each well containing the in 3D collagen matrix embedded cells was imaged with an LSM710 using an EC Plan-Neofluar DICI 10x/0.3 NA objective lens (Carl Zeiss). The stage was automated to acquire a 5×5 tile z-stack at each well starting from its very middle. The z-stack was set to begin 100 µm above and to end 500 µm below the surface of the 3D collagen gel. The settings for the LSM were as follows: zoom = 0.7, pixel dwell time = 1.58 µs, average = 1, master gain = 564, digital gain = 1.24, digital offset = −50.00, pinhole = 265 µm, filters = 410–585, laser line 405 nm = 4%. The acquired data sets were imported into Imaris 7.4.0 software (Bitplane) and cropped in 3D. 3D cropping is crucial as only the central part of the surface of the collagen matrix is flat, whereas in the periphery the surface is curved due to the formation of a meniscus. Then we applied the spot detection algorithm of the Imaris 7.4.0 software to assign a spot for each fluorescent intensity of a single nucleus. The settings for the spot detection algorithm were as follows: [Algorithm] enable region of interest = false; enable region growing = false; enable tracking = false. [Source channel] source channel index = 1; estimated diameter = 15; background subtraction = true. [Classify spots] “quality” above 3.089. Thus, by using Imaris’ statistical analysis tool, we were able to retrieve the total number of spot objects. Subsequently, the spot objects were filtered by their z-position (“position z” below a threshold in µm), whereas the threshold was set at the lowest point of the still visible but otherwise nearly flat meniscus. Thus we could select all spot objects that were invading the 3D collagen matrix, duplicate this selection to a new “spots object” and determine the number of invading spots by using Imaris’ statistical analysis tool. Finally, by correlating the number of spots representing the invading cells to the total number of spots, we determined the percentage of invading cells in the 3D collagen matrix. The maximal invasion depth was determined by using Imaris’ statistical analysis tool. Here, the difference between the z-position of the lowest non-invading cell and the deepest invading cell in the data set was measured.

### Cell Shape, Imaging and Data Analysis

MLg fibroblasts were transfected with an EGFP-N2 (Clontech) vector as described above. Then, cells were seeded in either conventional 2D cell-culture plastic dishes or on top of a 3D collagen matrix prepared as described above and incubated 72 hours for invasion. The cells in the 3D collagen gel were PBS washed and fixed in 4% paraformaldehyde (PFA) in PBS for 1 hour. After fixation confocal z-stacks were acquired with an LSM 710 using an LD Plan-Apochromat 25×/0.8 NA objective lens (Carl Zeiss). The settings for the LSM were as follows: zoom = 1.0, pixel dwell time = 1.58 µs, average = 2, master gain = 479, digital gain = 1.00, digital offset = 0.00, pinhole = 90 µm, filters = 410–495, laser line 405 nm = 4% and laser line 488 nm = 4%. The confocal fluorescent z-stacks were volume rendered with Imaris 7.4.0 software (Bitplane) and its statistical analysis tool was used for the readout of cell shape, (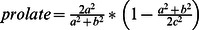, 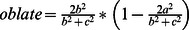, where a, b and c are the lengths of the semi-axes of an ellipsoid), cell surface area, cell volume and sphericity.

### Reagent Diffusion through a 3D Collagen Matrix

For testing the diffusion of reagents of various sizes through a 3D collagen matrix, we used a six channel μ-slide (IBIDI) and filled each channel with collagen prepared as described above, whereas the reservoir tanks of the channels were left empty. For gelation, the collagen filled µ-slide was incubated at 37°C for 3 hours and the quality of the polymerized collagen gels was checked with an Axiovert 40 C microscope (Zeiss). The reservoir tanks on both sides of three channels of the µ-slide were filled with 200 µl solution of Fluoresceinisothiocyanat (FITC) (Thermo Scientific) and a goat-anti-mouse IgG-Alexa568 antibody (Jackson ImmunoResearch Laboratories, Inc.). The reservoir tanks of the remaining three channels of the µ-slides were filled with 200 µl of Rhodamine Phalloidin (Life Technologies) and a goat-anti-mouse IgG-Alexa488 antibody (Jackson ImmunoResearch Laboratories, Inc.). Each of the reagents was diluted 1∶20 in PBS. Subsequently, time-series were acquired with an LSM 710 using an EC Plan-Neofluar 10×/0.30 NA objective lens (Carl Zeiss). Frames were acquired in intervals of 5 minutes for 7 hours. The settings for the LSM were as follows: zoom = 0.6, pixel dwell time = 1.58 µs, average = 1, master gain = 794, digital gain = 1.00, digital offset = 0.00, pinhole = 599 µm, filters = 410–495, laser line 488 nm = 2% and laser line 561 nm = 2%. For measuring the average grey values of each frame we used the open source software ImageJ (http://rsb.info.nih.gov/ij/; W. S. Rasband, NIH, National Institutes of Health, Bethesda, MD).

### Immunocytochemistry and Confocal Fluorescence Microscopy in 3D Collagen

MLg fibroblasts were seeded on top of the 3D collagen matrix, incubated, fixed and permeabilized as described above. Primary antibodies were diluted in 1% bovine serum albumin (BSA, Sigma) in PBS, incubated for 16 hours at 4°C and subsequently washed three times with PBS for 20 minutes each. Secondary antibodies were diluted in 1% bovine serum albumin (BSA, Sigma) in PBS, incubated for 16 hours at 4°C and subsequently washed three times with PBS for 20 minutes each. Cells were imaged in PBS with an LSM 710 as z-stacks and with an LD C-Apochromat 40×/1.1 NA water objective lens (Carl Zeiss). The settings for the LSM were as follows: zoom = 1.7, pixel dwell time = 2.55 µs, average = 4, master gain = 593, digital gain = 1.00, digital offset = 0.00, pinhole = 90 µm, filters = 410–495, laser line 488 nm = 10%, laser line 405 nm = 4% and laser line 561 nm = 2%.

### Separation Assay, Protein and mRNA Isolation

The separation assay is a modified setup of the invasion assay. Gelation of the 3D collagen gel was performed as described above. The 3D collagen matrix was directly put on the bottom side of tissue culture inserts for 6-well plates (ThinCerts™, 8 µm pore size, Greiner Bio-One). After gelation of the 3D collagen gel 5×10^5^ cells per well were seeded on top of the insert membrane and left for invasion under standard conditions (37°C, 5% CO_2_) in DMEM/HAM’s F12 medium containing 1% FBS for 72 hours. The tissue culture insert, containing membrane and 3D collagen gel, was washed twice with ice-cold PBS. Subsequently, the gel was separated from the membrane with a pair of tweezers. For protein isolation a minimum of three gels was pooled in one 2 ml Eppendorf tube and the remaining PBS aspirated. 80 µl (2120 U) of collagenase type1 (Biochrome) was added to each tube and incubated shaking at 37°C for 30–60 minutes until the complete disintegration of the collagen gel. Centrifugation for 2 minutes at 500 g at 4°C resulted in a cell pellet that was washed twice with ice-cold PBS. Finally, the cell pellet was lysed in 50 µl ice-cold RIPA buffer (50 mM Tris-Cl pH 7.4, 150 mM NaCl, 1% NP40, 0.25% Na-deoxycholate) containing 1× Roche complete mini protease inhibitor cocktail. For protein isolation from non-invading cells the membranes were cut out with a sharp scalpel. Next, the cells were scraped off the membrane directly into 200 µl ice-cold RIPA buffer containing 1× Roche complete mini protease inhibitor cocktail. Cells of a minimum of three membranes were pooled in one 2 ml Eppendorf tube. After incubating the samples for 30 minutes on ice, insoluble material was removed by centrifugation at 14.000 g for 15 minutes at 4°C and the supernatant was further processed.

For RNA isolation gel and membrane were separated as mentioned above. Gels were directly pooled in 1 ml of QIAzol Lysis Reagent (Qiagen), incubated for 10 minutes at room temperature and pipetted up and down until the complete disintegration of the collagen gel. For the membranes, a minimum of three was pooled in one well of a 6-well plate and incubated in 1 ml of QIAzol Lysis Reagent for 10 minutes. Then, each sample was transferred into a 1.5 ml Eppendorf tube and 200 µl of chloroform was added. After mixing, the phases of the samples were separated by centrifugation at 12000 g for 15 minutes at 4°C. The upper aqueous phase was transferred into a fresh tube and RNA was further purified with RNeasy Mini Kit (Qiagen) according to the manufacturer’s instructions. Centrifugation steps were performed with a Mikro200R table centrifuge (Hettich).

### SDS-Page, Western Blot and Densitometric Analysis

Samples were mixed with 50 mM Tris-HCl, pH 6.8, 100 mM DTT, 2% SDS, 1% bromphenol blue, and 10% glycerol, and proteins were separated using standard SDS-10% PAGE. For immunoblotting, proteins were transferred to nitrocellulose membranes, which were blocked with 5% milk in TBST (0.1% Tween 20/TBS) and incubated with primary, followed by HRP-conjugated secondary antibodies.

### cDNA-Synthesis and qRT-PCR Analysis

cDNA was synthesized with the GeneAMP PCR kit (Applied Biosystems) utilizing random hexamers using 1 µg of isolated RNA for one reaction. Denaturation was performed in an Eppendorf Mastercycler with the following settings: lid = 45°C, 70°C for 10 minutes and 4°C for 5 minutes. Reverse transcription was performed in an Eppendorf Mastercycler with the following settings: lid = 105°C, 20°C for 10 minutes, 42°C for 60 minutes and 99°C for 5 minutes. qRT-PCR reactions were performed in triplicates with SYBR Green I Master in a LightCycler® 480II (Roche) with standard conditions: 95°C for 5 min followed by 45 cycles of 95°C for 5 s (denaturation), 59°C for 5 s (annealing) and 72°C for 20 s (elongation). Target genes were normalized to GAPDH expression. Mouse primer sequences were as follows: ATCCCTTGATGCCATTACCA (MMP13_f), AAGAGCTCAGCCTCAACCTG (MMP13_r), TGTGTCCGTCGTGGATCTGA (GAPDH_f), CCTGCTTCACCACCTTCTTGA (GAPDH_r), CTCTGAGGCGTTTGGTGCTCCG (CXCR4_f), TGCAGCCGGTACTTGTCCGTC (CXCR4_r), AGGAGCTACTGACCAGGGAGCT (FSP-1_f), TCATTGTCCCTGTTCTGTCC (FSP-1_r).

### Statistics

We performed statistical analysis using GraphPad Prism4 (GraphPad Software). Data are presented as mean ± s.d. Statistical analysis was performed using unpaired and paired *t*-tests (two-tailed), and one-way Anova (non-parametric Kruskal-Wallis test) including Dunn’s multiple comparison post-test (α-level = 0.05).

## Results

**Figure 1:**
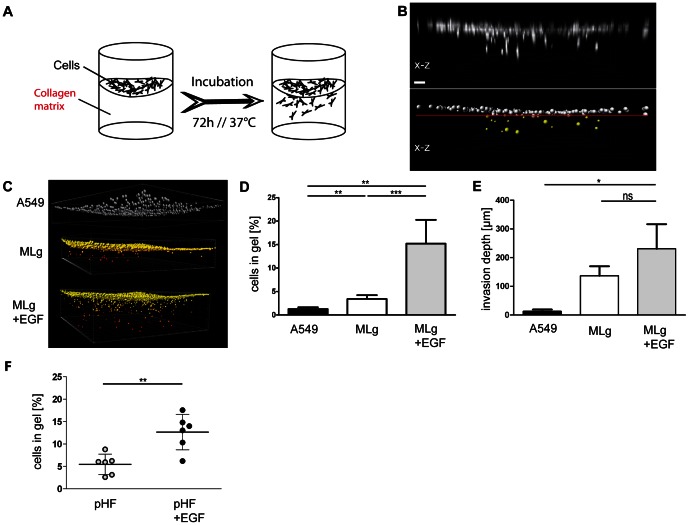
Multiplex profiling of invading MLg fibroblasts. A. Schematic representation of one well from a 96-well-plate used for the invasion assay and filled with an approximately 300 µm thick 3D collagen matrix. MLg fibroblasts were seeded on top of the collagen matrix and left for invading the matrix at 37°C for 72 h. B. Maximum intensity projection (x-z) from a z-stack taken with a confocal laser scanning microscope from DAPI stained MLg fibroblasts that either invaded the 3D collagen matrix or stayed on top (top panel). The bottom panel shows a spot analysis done with the Imaris (Bitplane) software of the z-stack shown in the upper panel. Invaded fibroblasts (above the red line) are depicted as yellow and non-invaded ones (below the red line) as grey spheres. Scale bar, 50 µm. C. Spot analysis of an invasion assay using A549 epithelial cells as non-invading cells (top panel, grey spheres) and MLg fibroblasts either untreated (middle panel) or treated with 50 ng/ml EGF (bottom panel). Invasion depth is color coded from yellow (non-invaded) to red (invaded). D. Quantitation and statistical evaluation of the amount of MLg fibroblasts that invaded the 3D collagen gel. Data shown represent mean values (± s.d.) from at least three independent experiments (A549: n = 3, CCL206: n = 5). **p<0.01 and ***p<0.001. E. Quantitation and statistical evaluation of the invasion depth of MLg fibroblasts. Data shown represent mean values (± s.d.) from three independent experiments (n = 3). *p<0.05, **p<0.01 and ns = not significant. F. Quantitation and statistical evaluation of the amount of primary human fibroblasts (pHF) that invaded the 3D collagen gel. Data shown represent mean values (± s.d.) from fibroblasts isolated from biopsies/resections of COPD patients (n = 6). **p<0.01.

Here, we present a validated 96-well microplate-based, 3D cell culture assay to assess cellular invasion in a high-throughput and high-content setting. 40 µl of collagen type I matrices (3.2 mg/ml) including 2% FBS were poured into each well of a black 96-well imaging plate (BD Biosciences) and allowed to polymerize. With the assistance of a laser scanning microscope (LSM 710) operating in reflection mode, the thickness of the polymerized 3D-collagen matrix measured 200–300 µm (*data not shown*). The quality of the collagen gel scaffold microstructure was found to be homogenous throughout the gel (Fig. S1 and Movie S1 and S2). By using a maximum of 300 µm thick collagen matrices in combination with thin-bottomed 96-well imaging plates, we were able to image cells on top of the collagen matrix not only with a 10×, but also with a 20× (LD), 25× (LD, Water), and 40× (LD, Water) objective. After gelation of the collagen matrix, lung fibroblasts (MLg, 2×10^4^/well) were seeded on top of the matrix and allowed to invade at 37°C for 72 hours in growth medium (Fig. 1A). After fixing and staining the cells’ nuclei with DAPI, confocal z-stacks were acquired for each well with an LSM 710 (Fig. 1B). The stage of the microscope was automated using ZEN2009 (Zeiss) software for a 96-well carrier, thereby acquiring one z-stack per well. In order to image a larger area of the gel, we acquired a tile-scan of 5×5 images/well. By applying these settings, scanning of 96 wells was carried out within one hour. For analysis, imaging data was imported into Imaris software (Bitplane). Importantly, since the small wells of a 96-well plate gave rise to a meniscus in the collagen matrix, we used the 3D-crop option of Imaris to use only the planar portion of the gel for analysis (Fig. 1B). Then, we applied the built-in spot detection algorithm in Imaris to assign one spot to each fluorescent intensity of a single nucleus and thus to a cell (Fig. 1B). As such, we were able to extract the exact spatial information about the position (x,y,z) of each single cell within the collagen matrix. Finally, we made use of Imaris’ statistical analysis function for spot-objects in order to differentiate between the amount of cells above (white spots) and below (yellow spots) a certain threshold in the z-direction (Fig. 1B). Setting of the threshold is crucial and was routinely set to the lowest point of the visible meniscus (see red line in Fig. 1B). Generally, analysis of a complete 96-well plate was accomplished within one hour.

In order to exclude matrix artifacts as false positive signals of cells in the collagen gel, we chose the human alveolar basal epithelial cell line A549 as a negative control for invasion. Only about 1% of A549 cells were found approximately 15 µm within the collagen matrix (Fig. 1C–E). As cell nuclei have an average diameter of approximately 10 µm, we considered the emergence of A549 cells within 15 µm of the collagen matrix as invasion-negative.

Next, we investigated whether the invasion assay could be used quantitatively to assess the effect of well-characterized growth factors on fibroblast invasion, such as epidermal growth factor (EGF). EGF is known to induce tumor cell invasion [11] and affect fibroblast migratory characteristics in conventional cell culture and 3D-hydrogels [12], [13]. Treatment of MLg fibroblasts with EGF (50 ng/ml) significantly (p = 0.0009, unpaired t-test) increased the amount of cells invading the 3D collagen matrix (15.2±4.5%, mean ± s.d.), compared with untreated MLg fibroblasts (3.4±0.8%, mean ± s.d.) (Fig. 1C,D). While EGF significantly (p = 0.0009, unpaired t-test) increased the number of invading fibroblasts, the invasion depth was only substantially increased upon EGF treatment (Fig. 1E). Additionally, we measured the invasion capacity of primary human fibroblasts (pHF) isolated from biopsies or resections of COPD patients (n = 6). Again, EGF treatment significantly (p = 0.0012, paired t-test) augmented the amount of pHFs that invaded the 3D collagen gel (12.7±3.9%, mean ± s.d.) (Fig. 1F).

**Figure 2:**
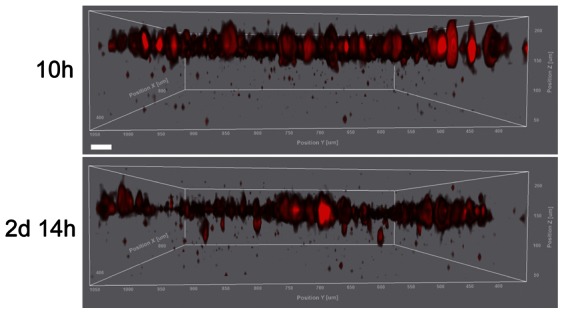
Live cell imaging of invading MLg fibroblasts. Maximum intensity projection image of two time points (10 hours and 2 days 14 hours, top and bottom panel, respectively) taken from a confocal 4D time-lapse movie. Fibroblasts were stained with the cell tracker dye CMTPX. Arrows in the bottom panel indicate invaded cells. Scale bar, 50 µm.

To further corroborate proper operation of our invasion assay, MLg fibroblasts were loaded with the cell tracker dye CMTPX, seeded on top of the 3D collagen matrix and analyzed by confocal 4D (z-stacks over time) time-lapse microscopy. Using this approach, we could live image MLg fibroblasts penetrating the 3D collagen matrix (arrows in Fig. 2 and Movie S3). MLg fibroblasts penetrated deeper into the 3D collagen gel over time and could be traced at an invasion depth of 98 µm after 2 days and 14 hours (Fig. S2).

**Figure 3:**
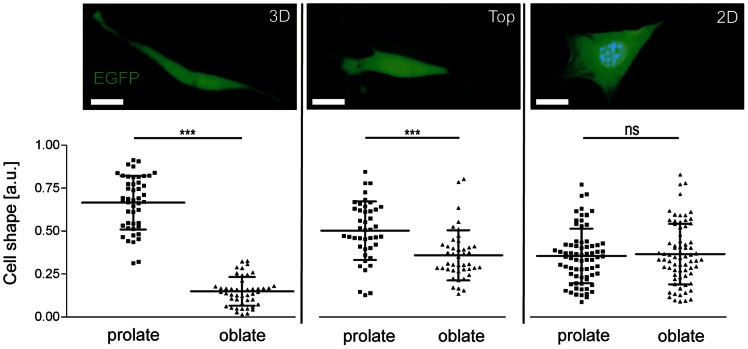
Morphological plasticity of MLg fibroblasts depends on the microenvironment. Representative confocal images, quantitation and statistical evaluation of cell shapes of MLg fibroblasts found either within (3D) or on top (Top) of the 3D collagen gel compared to fibroblastic cells cultured on conventional 2D plastic surfaces (2D). Data shown represent mean values (± s.d.) from randomly chosen cells (n = 47–73). ***p<0.001 and ns = not significant. Scale bar, 20 µm.

Cell morphology and function is known to be dependent on the immediate microenvironment surrounding the cells, such as ECM composition and rigidity [14], [15]. Therefore, we were interested to quantify cell morphology of MLg fibroblasts in conventional 2D culture compared with cells on top or within the 3D collagen matrix. MLg fibroblasts transfected with an EGFP-N2 vector were analyzed using confocal z-stacks that were volume- or surface-rendered with the Imaris software. In the numerical output, we observed a morphological switch of fibroblasts from rather disk-shaped spheroids (prolate = 0.36, oblate = 0.37) in conventional 2D cell culture to elongated cigar-shaped spheroids (prolate = 0.67, oblate = 0.15) in 3D collagen, thereby correlating to the flat (2D) and spindle-shaped (3D) cell morphology, respectively (Fig. 3). Accordingly, the cell surface area and volume of invaded (3D) MLg fibroblasts (A = 2254 µm^2^, V = 5291 µm^3^) were significantly (p<0.0001, one-way Anova) decreased when compared with 2D cultured cells (A = 2956 µm^2^, V = 9601 µm^3^). Non-invaded cells on top of the 3D collagen matrix showed the lowest surface area and volume (A = 1511 µm^2^, V = 4241 µm^3^) (Fig. S3 A,B). Additionally, MLg fibroblasts inside the 3D collagen matrix clearly showed a significant (p<0.0001, one-way Anova) lower sphericity (0.66) and thus a more elongated morphology than non-invaded (Top) (0.83) and 2D cultured cells (0.73) (Fig. S3C).

**Figure 4:**
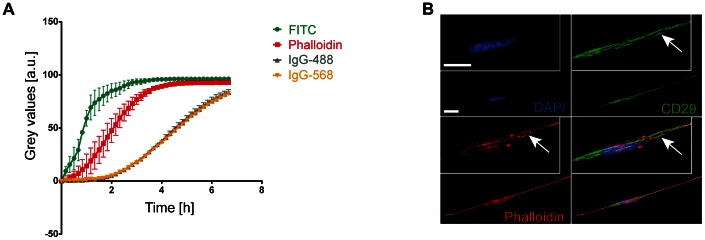
Diffusion of molecules and immunofluorescence staining in 3D collagen gels. A. Quantitation of the diffusion of FITC, Phalloidin, antibodies IgG-488 and IgG-568 over time. The fluorescent signals were measured by time lapse on a confocal laser scanning microscope. In the diagram the fluorescent signal is depicted as mean grey values (± s.d.) from three independent experiments (n = 3). B. Confocal image of one MLg fibroblast that invaded approximately 100–200 µm into the 3D collagen gel. Cells were stained for DAPI, Phalloidin and an antibody to CD29 (Integrin β1). Boxed areas show a magnified view of the central part of the spindle-shaped cell. The white arrow indicates an elongated fibrillar adhesion-like structure in the CD29 staining that co-localizes with actin stress fibers (Phalloidin). Scale bars, 20 µm.

Next, we sought to test whether invaded MLg fibroblasts could be stained with conventional immunofluorescence tools, considering that the dense network of the 3D collagen matrix may influence reagent diffusion parameters, particularly for high molecular weight molecules like antibodies. Protocols for immuno-labeling and high-resolution imaging of cells in three-dimensional collagen matrix were published before [16], though the diffusion of fluorescently-tagged antibodies throughout such matrices has not been measured up to now. Here, we assessed diffusion rates of fluorescent molecules of various chemical structure and size through a 3D collagen matrix by using time-lapse confocal microscopy. IgG-488 and IgG-596 antibodies exhibited a slow diffusion kinetic in the collagen matrix and did not reach full saturation even after 6 hours of testing. However, small molecules like FITC and Phalloidin were fully diffused after 4 hours (Fig. 4A and Fig. S4). Thus, immunofluorescence stainings of cells in 3D collagen gels require at least overnight incubation. Next, MLg fibroblasts that invaded 100–200 µm into the gel were stained with DAPI or Phalloidin, or with antibodies against the cell-matrix receptor integrin β1 (CD29), the nuclear proliferation-marker Ki67, the extracellular-matrix protein fibronectin, or the intermediate-filament protein vimentin. We could visualize specific staining of fibrillar adhesion-like structures along stress fibers with the CD29 antibody (Fig. 4B), nuclear staining of Ki67, staining of fibronectin fibrils, and intracellular staining of vimentin filaments of invaded MLg fibroblasts (Fig. S5). Additionally, by reversing the 3D collagen matrix upside down onto a coverslip, we were further able to image cells that invaded the gel to depths of 100 µm with high-resolution 63× and 100× objectives (*data not shown*).

**Figure 5:**
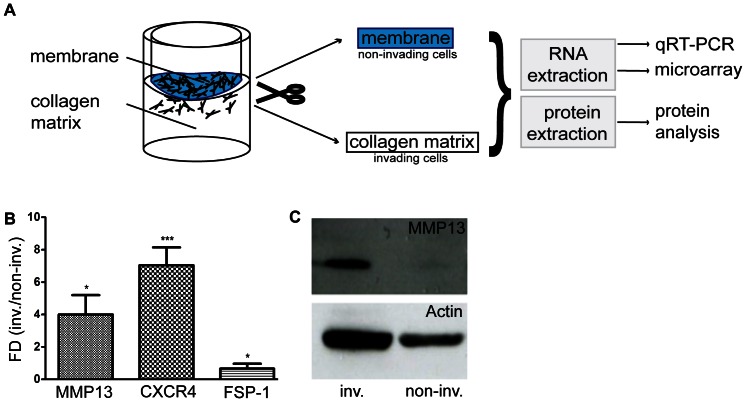
Molecular profiling of invading MLg fibroblasts. A. Scheme of a modified invasion assay using a porous membrane (depicted in blue color) of cell culture inserts to separate invading from non-invading MLg fibroblasts. Fibroblasts were seeded on top of the collagen matrix and left for invading the matrix at 37°C for 72 h. Separation of membrane and collagen matrix is followed by RNA and protein extraction with subsequent qRT-PCR, gene-microarray and protein analysis. B. Differential mRNA expression analysis (qRT-PCR) of MMP13, CXCR4 and FSP-1 in invasive MLg fibroblasts. Data shown represent the fold difference (FD = 2^ΔΔCp^) between invading (inv.) versus non-invading (non-inv.) MLg fibroblasts. Data shown represent mean values (± s.d.) from three independent experiments (n = 3). *p<0.05 and ***p<0.001. C. Representative Western blot of cell lysates comparing MMP13 levels from invading (inv.) versus non-invading (non-inv.) MLg fibroblasts.

Next, we sought to analyze the invasive properties of fibroblasts on a molecular level. We therefore modified our invasion assay by employing a porous membrane of cell-culture inserts in combination with a 3D collagen matrix to physically separate invaded from non-invaded MLg fibroblasts. After 72 h of incubation at 37°C, the membranes containing non-invaded cells were detached from the 3D collagen matrix containing the invaded cells. Subsequently, we performed protein or mRNA extraction and used the samples for protein analysis or qRT-PCR/microarray, respectively (Fig. 5A). mRNA levels of MMP13, a key regulator of cellular invasion [17], were significantly (p = 0.0345, paired t-test) augmented in invaded fibroblasts (4±1.2 fold, mean ± s.d.) (Fig. 5B). We also demonstrated by qRT-PCR that the chemokine receptor CXCR4, that was shown to have a rather low half-life of 1.4 hours in mouse embryonic stem cells [18], showed a significant (p = 0.0003, paired t-test) increase in mRNA levels of 7.0±1.1 fold (mean ± s.d.). Fibroblast-specific protein-1 (FSP-1), whose mRNA reportedly has a half-life of 12.7 hours [18] was found to be significantly (p = 0.04, paired t-test) down regulated by 0.7±0.3 fold (mean ± s.d.). Likewise, in a microarray analysis we could observe a similar deregulation of mRNA levels of before mentioned targets (*data not shown*). For MMP13 these data were corroborated on protein level (Fig. 5C).

## Discussion

Here, we have developed a versatile toolbox for extensive high-throughput profiling of invasive cell types in a real 3D cell culture setup based on 96-well plates. Our technique is applicable to a wide spectrum of cells ranging from different pathological and physiological origins, either as cell lines or primary cell types. This also includes cells from knockout or transgenic animals. We argue that our large-scale assay is apt for mechanistic studies of aberrant motility processes of cells harvested from diseases like fibrosis, metastatic cancers, or chronic inflammatory states.

Commercially available invasion assays like the Oris™ (Platypus) cell invasion assays work on 96-well basis and were successfully applied by Freytag et al. [19]. These assays use seeding stoppers creating a cell exclusion zone that is subsequently overlaid with collagen I or any other component of extracellular matrix. While this assay undoubtedly has a number of strengths, this technique rather has the features of a scratch assay monitoring cells that migrate into the cell exclusion zone. Subsequently, the closure of the exclusion zone is measured. In our view, there is no real control whether cells really invade the overlaid ECM or rather migrate along the plastic bottom merely closing the cell free area.

Our method is easily expandable, as using 3D collagen gels of altered stiffness, mechanical or chemical gradients, or modulating the ECM by specifically adding components like distinct collagens, laminins, or elastin will add another layer of versatility in the readout. Modulation of the ECM might become a seminal issue in the future, as the active role of a remodeled ECM in the progression of fibrotic events has recently been highlighted [20]. In the future, antifibrotic therapies might be based on targeting specific components of the ECM and/or enzymes modifying these ECM components [20], [21]. Proteomic analysis of ECM components in a high-throughput setting of 3D collagen gels, that were penetrated by invading cells derived from healthy and diseased tissues, might further shed light on aberrant invasive motility processes. Even more advanced, analysis of differential protein expression in the ECM has been performed in vivo for lung fibrosis [22] and cartilage development [23]. Furthermore, as the 3D collagen matrix proved to be penetrable to large molecules, our approach is also applicable for testing antibodies and/or recombinant proteins tackling migratory processes. Additionally, the assay described here does not require specialized equipment above a standard laser-scanning microscope, a powerful workstation, and a software analysis tool such as Imaris (Bitplane). In the future, a slightly modified version of the assay will be implemented to assess various parameters of 3D live migration, including speed or directionality by applying high-throughput 4D live imaging. Analysis of 3D live migration data can easily be batch processed in Imaris (Bitplane) to significantly increase the analysis speed of data sets, thereby making it ideal for the primary screening of siRNAs or chemical compounds. An additional miniaturization to 384 wells and robotic processing should further extend the overall screening process.

Recently, an elegant method for studying chemotactic dynamics of cellular invasion has been published [24]. By using a custom-made incubation chamber for time-lapse microscopy, this chemotaxis assay can only address one condition at a time. Tracking of cells is performed on projected images of z-stacks, thus losing information about migration throughout the third dimension. Stable chemotactic gradients are hard to achieve with our invasion assay, though a gradient could be accomplished by casting various gels with different ligand concentrations on top of each other. On the contrary, such a complex gel setup might also increase the variation in the read out of the assay. Furthermore, by co-culturing cells, for instance epithelial cells on top of and fibroblasts within the gel, one might be able to establish a physiological chemotactic gradient assessing invasion of epithelial cells and fibroblasts alike.

Taken together, our multiplex profiling assay will enable the evaluation of a plethora of novel reagents involved in cellular migration in 3D cell culture systems and will be of high value for gathering new data in basic, translational, as well as pharmaceutical research alike.

## Supporting Information

Figure S1 **Microstructure of the 3D collagen gel.** Maximum intensity projections (x-y, x-z) from a z-stack that was taken with a confocal laser scanning microscope displaying the delicate fibers of the 3D collagen microstructure (grey). Collagen was imaged by operating the confocal laser scanning microscope in reflective mode. Cells were stained for DAPI (blue) and Phalloidin (red). Scale bar, 10 µm.
(TIF)

Figure S2 **4D live invasion imaging of MLg fibroblasts.** Single frames at different time points (10 hours, 1 day 7 hours, 1 day 22 hours, 2 days 14 hours) taken from a 4D time-lapse movie of MLg fibroblasts that were stained with the cell tracker dye CMTPX. White arrows in the different panels indicate cells that penetrated into deeper regions of the 3D collagen matrix over time. Scale bar, 80 µm.
(TIF)

Figure S3 **Assessing morphological properties of invading MLg fibroblasts.** Quantitation and statistical evaluation of cell surface area (µm^2^), cell volume (µm^3^) and cell sphericity (a.u.) from MLg fibroblasts found either within (3D) or on top (Top) of the 3D collagen gel compared to cells cultured on conventional 2D plastic surfaces (2D). Data shown represent mean values (± s.d.) from randomly chosen cells (n = 47–73). *p<0.05, **p<0.01, ***p<0.001 and ns = not significant.
(TIF)

Figure S4 **Diffusion of molecules through 3D collagen gels.** The image displays selected frames at different time points from a time lapse movie assessing fluorescent signals of FITC, Phalloidin, antibodies IgG-488 and IgG-568 diffusing a 3D collagen gel in an IBIDI µ-slide. Images were taken as a time lapse by confocal laser scanning microscopy measuring the fluorescence signals over time.
(TIF)

Figure S5 **Immunofluorescence staining in 3D collagen gels.** Immunofluorescence confocal microscopy (in green: Ki67, fibronectin and vimentin) of invaded MLg fibroblasts counterstained with DAPI (blue) and Phalloidin (red). The images are displayed as maximum intensity projections from confocal z-stacks. The Ki67 staining clearly shows specific nuclear staining (white arrows in the green and merged channel of the top panel). Scale bar top panel, 20 µm. Scale bar middle and bottom panel, 10 µm.
(TIF)

Movie S1 **Microstructure of the 3D collagen gel.** The movie shows a fly-through from the top of a confocal z-stack to its bottom. The slices display the delicate fibers of the 3D collagen microstructure at different positions along the z-axis. Collagen (grey) was imaged by operating the confocal laser scanning microscope in reflective mode. Cells were stained for DAPI (blue) and Phalloidin (red). Scale bar, 10 µm.
(MOV)

Movie S2 **Invaded lung fibroblast embedded in a 3D collagen matrix.** 360° rotation of a 3D volume rendered (maximum intensity projection) confocal z-stack shown in Fig. S1 and Movie S1. Collagen (grey) was imaged by operating the confocal laser scanning microscope in reflective mode. Cells were stained for DAPI (blue) and Phalloidin (red).
(MOV)

Movie S3 **4D invasion assay.** Confocal 4D (z-stacks over time) time-lapse movie that was rendered as a maximum intensity projection with Imaris software. Each large graphical object depicted in red represents one cell whereas single cells are observed to invade 50–100 µm deep into the 3D collagen gel (see also Fig. 2 and Fig. S2). Small speckle-like objects, that are visible throughout the whole movie, represent background noise due to image acquisition. MLg lung fibroblasts were stained with the cell tracker dye CMTPX. Recording started within one hour after seeding the cells on top of the 3D collagen matrix and z-stacks were acquired in 30 minutes intervals. Scale bar, 70 µm.
(MOV)
