# Supplementary figures and images for: Multiplex Profiling of Cellular Invasion in 3D Cell Culture Models
Source: PLoS One. 2013 May 9;8(5):e63121. doi: 10.1371/journal.pone.0063121 (PMC3650046; doi:10.1371/journal.pone.0063121)

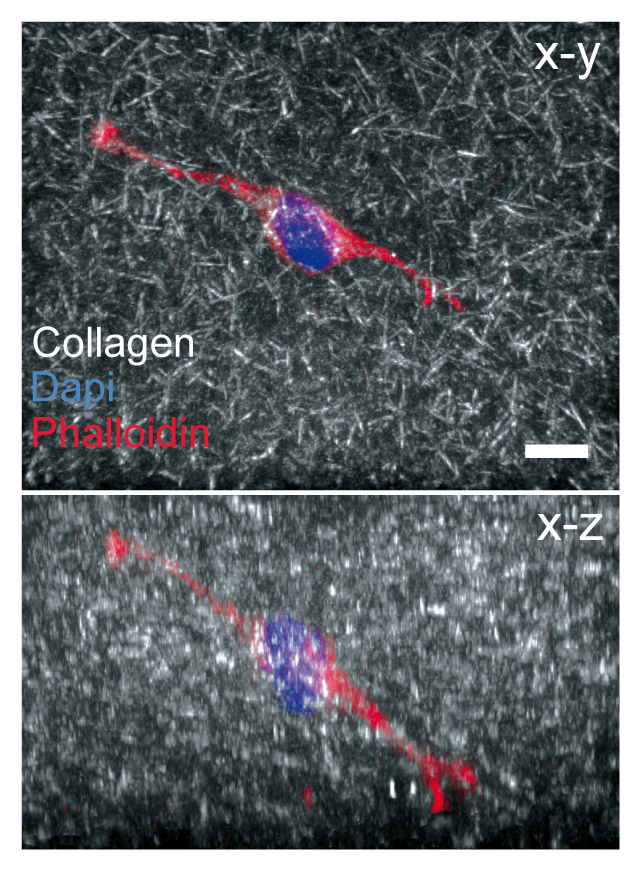

Supplement: Figure S1 — Microstructure of the 3D collagen gel. Maximum intensity projections (x-y, x-z) from a z-stack that was taken with a confocal laser scanning microscope displaying the delicate fibers of the 3D collagen microstructure (grey). Collagen was imaged by operating the confocal laser scanning microscope in reflective mode. Cells were stained for DAPI (blue) and Phalloidin (red). Scale bar, 10 µm. (TIF) [file pone.0063121.s001.tif]

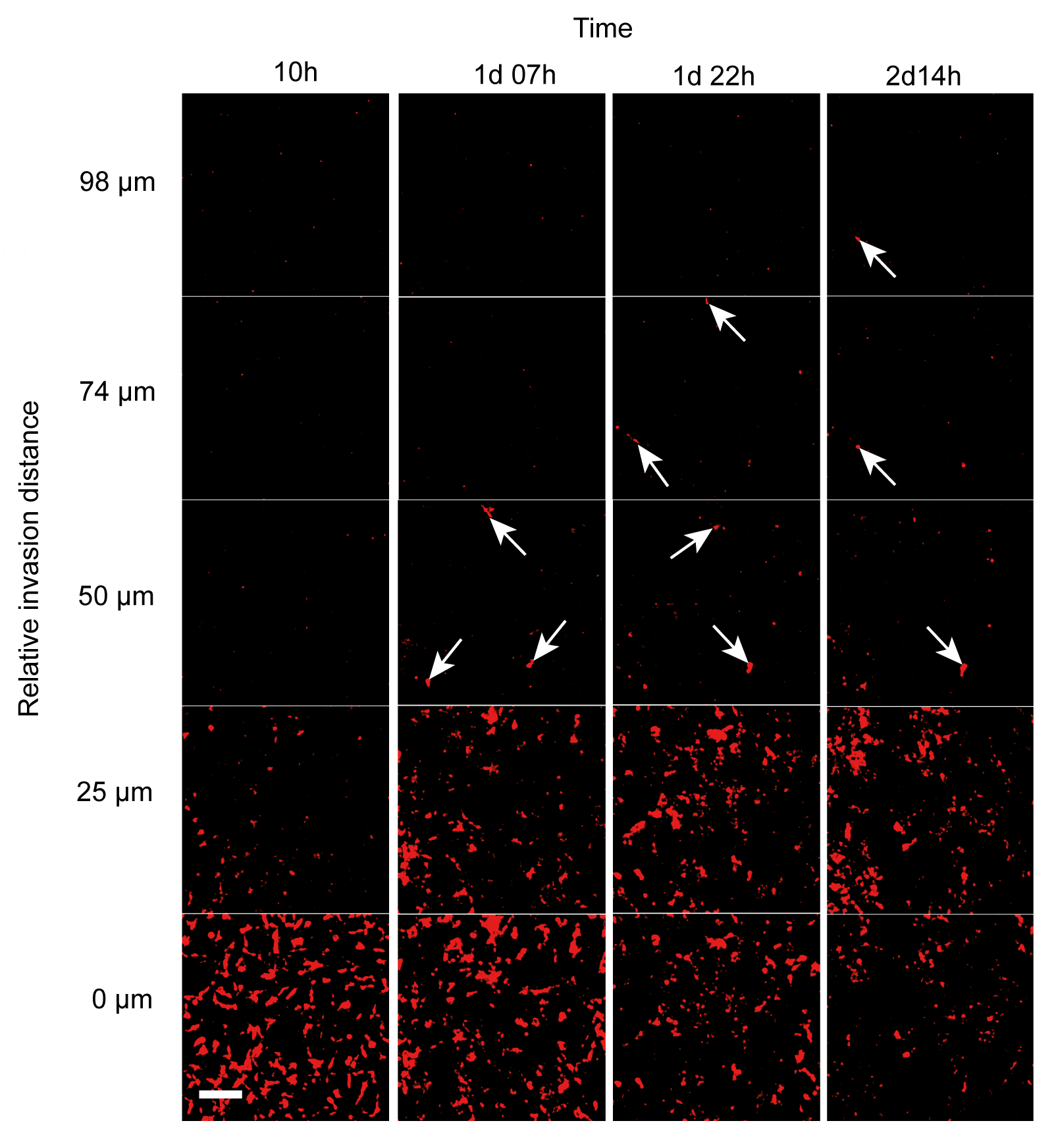

Supplement: Figure S2 — 4D live invasion imaging of MLg fibroblasts. Single frames at different time points (10 hours, 1 day 7 hours, 1 day 22 hours, 2 days 14 hours) taken from a 4D time-lapse movie of MLg fibroblasts that were stained with the cell tracker dye CMTPX. White arrows in the different panels indicate cells that penetrated into deeper regions of the 3D collagen matrix over time. Scale bar, 80 µm. (TIF) [file pone.0063121.s002.tif]

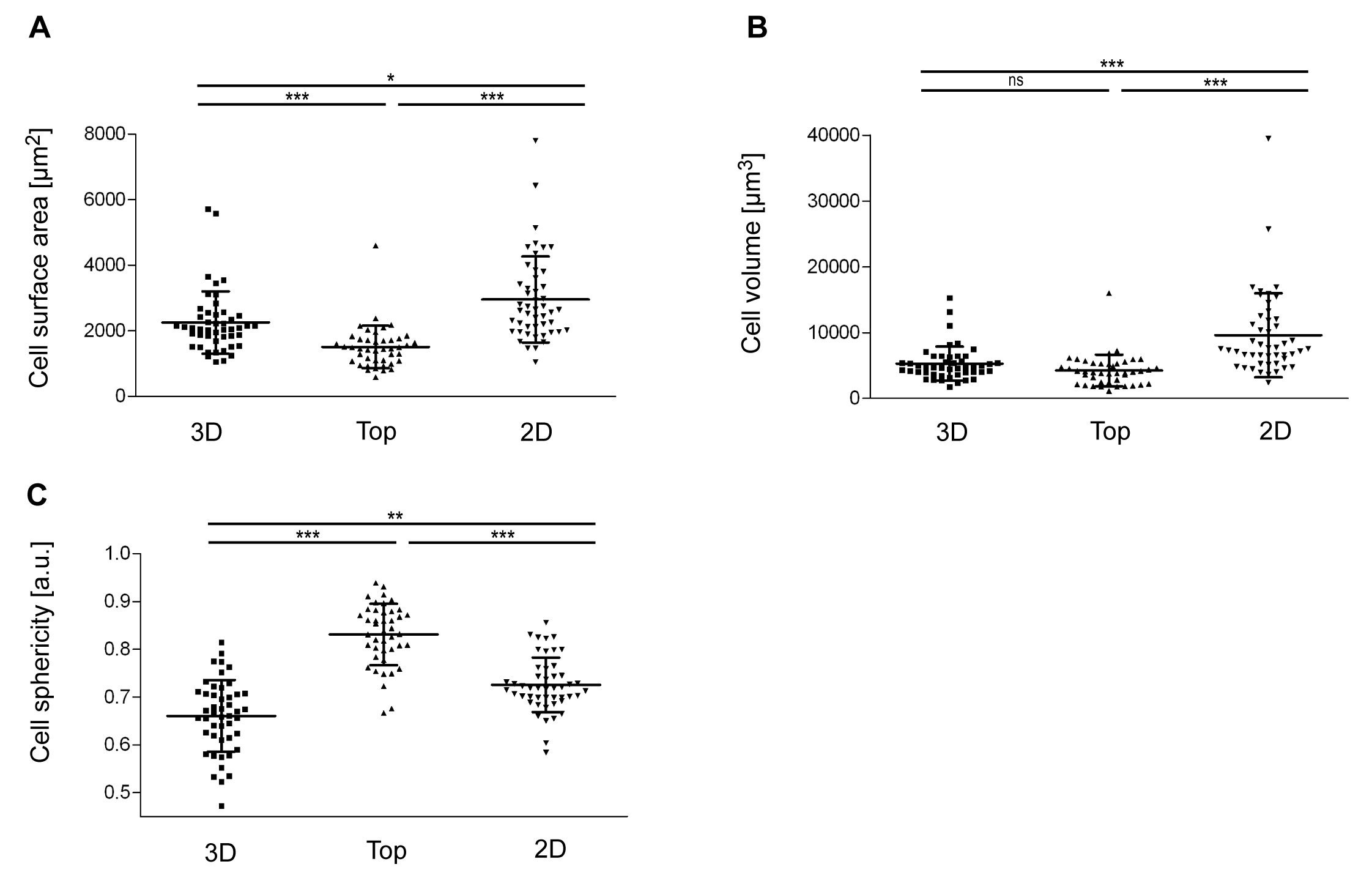

Supplement: Figure S3 — Assessing morphological properties of invading MLg fibroblasts. Quantitation and statistical evaluation of cell surface area (µm2), cell volume (µm3) and cell sphericity (a.u.) from MLg fibroblasts found either within (3D) or on top (Top) of the 3D collagen gel compared to cells cultured on conventional 2D plastic surfaces (2D). Data shown represent mean values (± s.d.) from randomly chosen cells (n = 47–73). *p<0.05, **p<0.01, ***p<0.001 and ns = not significant. (TIF) [file pone.0063121.s003.tif]

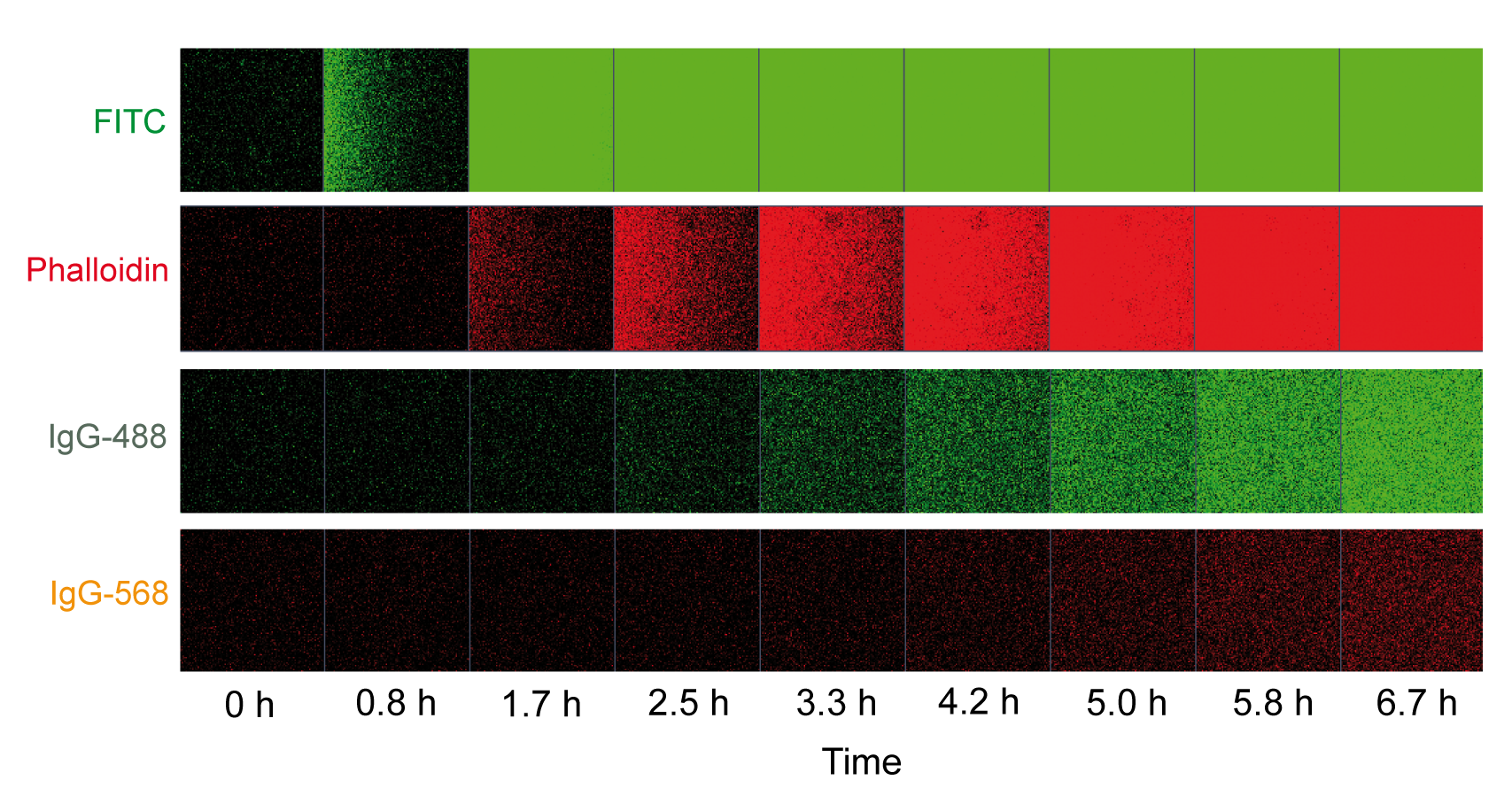

Supplement: Figure S4 — Diffusion of molecules through 3D collagen gels. The image displays selected frames at different time points from a time lapse movie assessing fluorescent signals of FITC, Phalloidin, antibodies IgG-488 and IgG-568 diffusing a 3D collagen gel in an IBIDI µ-slide. Images were taken as a time lapse by confocal laser scanning microscopy measuring the fluorescence signals over time. (TIF) [file pone.0063121.s004.tif]

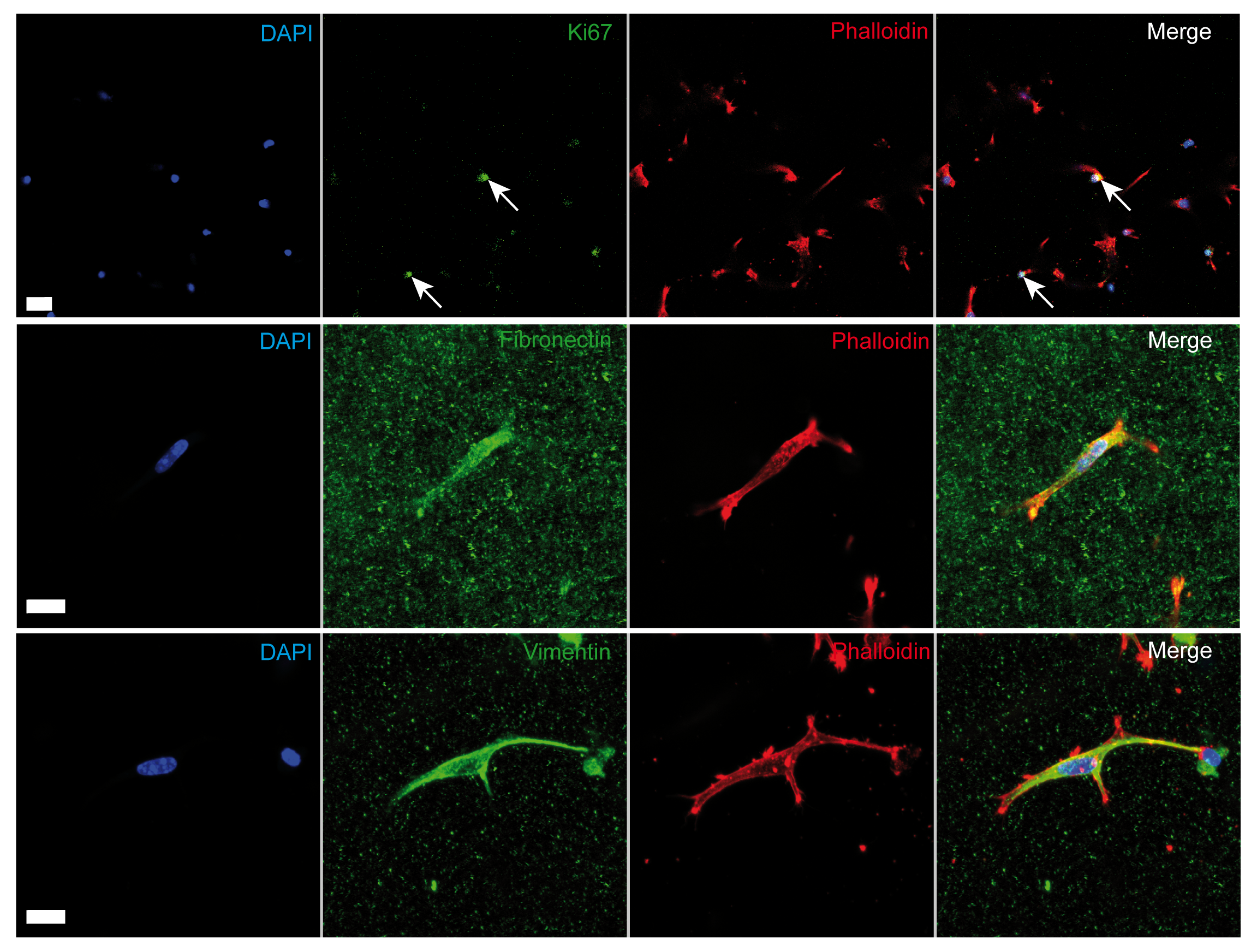

Supplement: Figure S5 — Immunofluorescence staining in 3D collagen gels. Immunofluorescence confocal microscopy (in green: Ki67, fibronectin and vimentin) of invaded MLg fibroblasts counterstained with DAPI (blue) and Phalloidin (red). The images are displayed as maximum intensity projections from confocal z-stacks. The Ki67 staining clearly shows specific nuclear staining (white arrows in the green and merged channel of the top panel). Scale bar top panel, 20 µm. Scale bar middle and bottom panel, 10 µm. (TIF) [file pone.0063121.s005.tif]
